# Surgical Delay-Associated Mortality Risk Varies by Subtype in Loco-Regional Breast Cancer Patients in SEER-Medicare

**DOI:** 10.21203/rs.3.rs-4171651/v1

**Published:** 2024-04-08

**Authors:** Macall Leslie, Rashmi Pathak, William C. Dooley, Ronald A. Squires, Hallgeir Rui, Inna Chervoneva, Takemi Tanaka

**Affiliations:** University of Oklahoma Health Sciences Center, Stephenson Cancer Center, 975 NE, 10th, Oklahoma City, OK 73104, USA; University of Oklahoma Health Sciences Center, Stephenson Cancer Center, 975 NE, 10th, Oklahoma City, OK 73104, USA; University of Oklahoma Health Sciences Center, School of Medicine, Dept. of Surgery, 800 Stanton L. Young Blvd., Oklahoma City, OK 73104, USA; University of Oklahoma Health Sciences Center, School of Medicine, Dept. of Surgery, 800 Stanton L. Young Blvd., Oklahoma City, OK 73104, USA; Thomas Jefferson University, Department of Pharmacology and Experimental Therapeutics, 1015 Chestnut St., Suite 520, Philadelphia, PA 19107, USA; Thomas Jefferson University, Division of Biostatistics, Department of Pharmacology and Experimental Therapeutics, 1015 Chestnut St., Suite 520, Philadelphia, PA 19107, USA; University of Oklahoma Health Sciences Center, Stephenson Cancer Center, 975 NE, 10th, Oklahoma City, OK 73104, USA; University of Oklahoma Health Sciences Center, School of Medicine, Dept. of Pathology, 800 Stanton L. Young Blvd., Oklahoma City, OK 73104, USA

**Keywords:** Surgical delay, Breast cancer-specific mortality, SEER-Medicare, Tumor subtype, Hormone-receptor, HER2

## Abstract

Substantial evidence supports that delay of surgery after breast cancer diagnosis is associated with increased mortality risk, leading to the introduction of a new Commission on Cancer quality measure for receipt of surgery within 60 days of diagnosis for non-neoadjuvant patients. Breast cancer subtype is a critical prognostic factor and determines treatment options; however, it remains unknown whether surgical delay-associated breast cancer-specific mortality (BCSM) risk differs by subtype. This retrospective cohort study aimed to assess whether the impact of delayed surgery on survival varies by subtype (hormone [HR]+/HER2−, HR−/HER2−, and HER2+) in patients with loco-regional breast cancer who received surgery as their first treatment between 2010–2017 using the SEER-Medicare. Continuous time to surgery from diagnostic biopsy (TTS; days) in reference to TTS = 30 days. BCSM were evaluated as flexibly dependent on continuous time (days) to surgery from diagnosis (TTS) using Cox proportional hazards and Fine and Gray competing-risk regression models, respectively, by HR status. Inverse propensity score-weighting was used to adjust for demographic, clinical, and treatment variables impacting TTS. Adjusted BCSM risk grew with increasing TTS across all subtypes, however, the pattern and extent of the association varied. HR+/HER2− patients exhibited the most pronounced increase in BCSM risk associated with TTS, with approximately exponential growth after 42 days, with adjusted subdistribution hazard ratios (sHR) of 1.21 (95% CI: 1.06–1.37) at TTS = 60 days, 1.79 (95% CI: 1.40–2.29) at TTS = 90 days, and 2.83 (95% CI: 1.76–4.55) at TTS = 120 days. In contrast, both HER2 + and HR−/HER2− patients showed slower, approximately linear growth in sHR, although non-significant in HR−HER2−.

## Introduction

A recent meta-analysis found that treatment delay is a critical factor contributing to mortality risk in multiple types of solid tumors, including breast cancer^1^. Extended time to surgery from diagnostic biopsy (TTS) in particular has negative survival implications in breast cancer^2–6^ and studies have noted that both the frequency and length of delay are increasing^7–9^. Thus, in 2022 the Commission on Cancer (CoC) introduced a new quality measure for accredited facilities of receipt of surgery within 60 days of diagnosis for Stage I-III patients in the non-neoadjuvant setting^10^ in order to address the negative survival impact of surgical delay. Breast cancer subtype is a critical prognostic factor that provides critical therapy-relevant information, since the underlying biologic differences reflect tumor behavior as well as treatment options^11^. Thus, questions remain as to whether all patients are predisposed to an equal level of mortality risk posed by extended TTS or whether risk differs with intrinsic properties such as subtype. Only a few studies have explored outcomes in relation to TTS by subtype. In a retrospective study of 351,087 Stage I-III breast cancer patients, Mateo et al. reported a consistent 10% increase in risk of overall mortality each subsequent month after the first 30 days post-diagnosis in a cohort of 351,087 Stage I-III patients that did not change based on subtype^12^. In contrast, in a cohort of 90,405 T1N0 breast cancer patients who received breast conserving surgery, Hills et al. found that risk of TTS-associated disease progression was confined to only hormone receptor (HR) + disease, with 18% and 47% higher likelihood of tumor size progression for patients who waited between 61 and 90 days and over 90 days, respectively^13^. While research thus far has shed critical light on the steady increase of surgical delay among breast cancer patients^7–9^ and the risk it presents for mortality outcomes^1–3^, the commonly adopted approach of examining TTS as fixed monthly or bi-monthly increments hinders vital understanding of how breast cancer-specific mortality (BCSM) risk may flexibly change with increasing TTS by subtype. Thus, the key objective of this study was to gain a comprehensive picture of whether TTS differentially impacts BCSM by subtype through flexible modeling of daily estimates of risk in women with loco-regional breast cancer in the non-neoadjuvant setting using the Surveillance, Epidemiology, and End Results (SEER)-Medicare database.

## Methods

### Cohort

**Figure 1:**
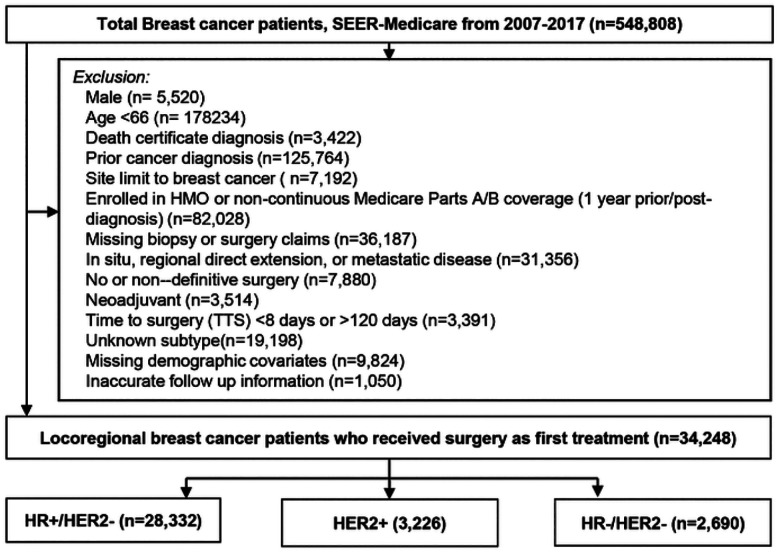
SEER-Medicare Exclusion Scheme

A retrospective cohort of women diagnosed by needle or incisional biopsy with loco-regional invasive, non-inflammatory breast cancer between 2010–2017 in the SEER-Medicare database who received surgery as their first treatment was selected. The SEER-Medicare linked database combines Medicare Parts A and B claims with clinical and outcome data from SEER cancer registries^14^. All data were de-identified and met the criteria for exempt review by the University of Oklahoma Health Sciences Center Institutional Review Board (IRB7446). Patients who had HMO coverage or did not have continuous Part A and B coverage for at least one year prior through one year after diagnosis were excluded due to the inability to accurately ascertain diagnostic or treatment claims for the primary course of treatment. Patients who received surgery within 7 days of diagnostic biopsy were excluded since the time required for pathologic molecular diagnosis commonly takes up to one week^15^. Additionally, patients that did not receive surgery until over ≥ 120 after diagnosis, had a time of death less than one year, SEER reported follow-up shorter than TTS, non-definitive initial surgery (i.e. re-excisions), prior cancer diagnosis, nonlocoregional disease (i.e. in situ, regional direct extension, or distant metastatic spread), or missing information were excluded (Fig. 1).

### Exposure

The primary exposure, time-to-surgery (TTS), was defined as days from date of diagnostic biopsy to date of surgery.

### Outcome

Breast cancer-specific mortality (BCSM) in the presence of competing events (i.e. death from other causes) was assessed and survival times were calculated from the date of surgery to death or last contact (censored).

### Definitions

The cohort was stratified by hormone receptor (i.e., estrogen and/or progesterone receptor; [HR]) and HER2 status into 3 groups: HR+/HER2−, HR−/HER2−, and HR + or HR-/HER2+ [HER2+]). Age at the time of diagnosis was categorized in 5-year intervals (i.e. <70, 70–74, 75–79, 80–84, and ≥ 85 years old). Race/ethnicity was categorized as non-Hispanic Black (Black), other (Asian, Hispanic, Pacific Islander, American Indian/Eskimo/Aleutian), or non-Hispanic White (White). The Charlson Comorbidity Index was calculated for each patient using the SEER-Medicare developed Comorbidity SAS Macro (2021 version) to search for relevant claims in the year prior to diagnosis, and classified as 0, 1, or ≥ 2. Education (% of residents without high school degree) and residential median income were based on census tract level information from the 2010 U.S. Census and the patient’s census tract of residence at the time of diagnosis. Histology was categorized as ductal, lobular, or other by ICD-O-3 codes (**Appendix 1**). The HCPCS, ICD-9, and ICD-10 codes used to classify diagnosis, surgery, and adjuvant therapies in the Medicare claims are listed in **Appendix 2.** Surgery type was classified as breast conserving, mastectomy, or mastectomy with immediate reconstruction.

### Statistical Methods

Time to death as a function of TTS was analyzed separately by subtype using Fine-Gray competing risk models for BCSM. All models were adjusted using inverse propensity score weights (IPW) to account for potential imbalances in covariates associated with TTS^16–18^. Covariate balancing propensity scores were computed using the R package “CBPS” with socio-demographic and clinical factors as predictors and log-transformed TTS as the response variable^18^. Pre-/post-weighting balance was assessed for each model using Love plots. Final survival models were adjusted by normalized IPW, with extreme weights beyond the 95th percentile winsorized, along with receipt of adjuvant radiation or systemic therapy, comorbidity score, and in HER2 + patients, hormone receptor status. B-splines were used to flexibly model the subdistribution hazard of mortality as nonparametric functions of TTS. Subdistribution hazard ratio (sHR) estimates were calculated using TTS = 30 days as the reference point, since it is commonly used as the upper limit of the reference in categorical TTS studies^2,6,12^. Simultaneous 95% confidence intervals (CI) at each TTS point were computed using the Scheffe method. The association between TTS and sHR was considered significant when the simultaneous 95% CI did not include a subdistribution hazard ratio of 1. To provide estimates of the BCSM incidence at TTS of 30, 60, 90, and 120 days, the adjusted cumulative incidence function was derived from the Fine-Gray model conditioned on the subgroup of patients with most the most common characteristics. All statistical analyses were conducted using SAS (version 9.4; Cary, NC) and R software (version 4.0.4), and graphs were generated using JMP Pro 15.2.0 (SAS; Cary, NC).

## Results

### Cohort characteristics.

Following exclusions (Fig. 1), 34,248 loco-regional breast cancer patients diagnosed between 2010–2017 who received as surgery first treatment (i.e., non-neoadjuvant) were selected from the SEER-Medicare database. The median age at diagnosis was 73 years old (range: 66 to 100 years old; first quartile Q1:69, third quartile Q3:78), and median follow-up time after surgery was 4.2 years (range: 0 days to 8.9 years; Q1: 2.4 years, Q3: 6.3 years). Approximately 82.7% (n = 28,332) of patients were HR+/HER2−, 9.4% (n = 3,226) were HER2+, and 7.9% (n = 2,690) were HR−/HER2−(Table 1). Median TTS was the same across subtypes (29 days) but ranged by clinical and demographic characteristics. Black patients had longer median TTS than White, particularly in HR+/HER2− (34 vs. 29 days) or HR−/HER2− (36 vs. 28 days). Additionally, median TTS increased steadily with year of diagnosis from 2010 to 2017, 6 and 7 days longer in HR+/HER2− and HER2+, respectively. Notably, the largest difference in median TTS was observed for patients who received mastectomy with immediate reconstruction (45 days in HR+/HER2−, 44 days in HER2+, and 41 days in HR−/HER2−).

### Pattern of BSCM risk associated with TTS varies by subtype.

**Figure 2:**
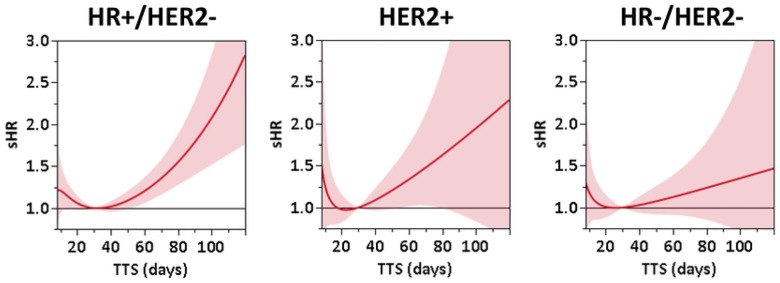
Adjusted risk (subdistribution hazard ratio [sHR]) of breast cancer-specific mortality (BCSM) associated with continuous TTS in HR+/HER2−, HER2+, and HR−/HER2− locoregional breast cancer patients in a SEER-Medicare cohort.

After inverse propensity score weight-adjustment, BCSM risk grew across all subtypes with increasing TTS, yet differing extent and patterns were noted by subtype (Fig. 2 and Table 2). In HR+/HER2−, the sHR exhibited approximately exponential growth starting at TTS = 42 days, equivalent to 10% higher risk each week relative to the one prior (**Supplemental Fig. 1**). Accordingly, adjusted sHR reached 1.21 (95% CI: 1.06–1.37) at TTS = 60 days, 1.79 (95% CI: 1.40–2.29) at TTS = 90 days, and 2.83 (95% CI: 1.76–4.55) at TTS = 120 days compared to the reference of TTS = 30 days (Fig. 2). For HER2 + patients, the sHR increased approximately linearly by 0.10 each week after TTS = 30 days (**Supplemental Fig. 1**). This increase was statistically significant for TTS in the range 55–85 days, but not for TTS > 85, presumably due to a small number of events resulting in wide confidence bounds. The sHR relative to TTS = 30 days were 1.34 (95% CI: 1.02–1.76) at TTS = 60 days, 1.78 (95% CI: 0.92–3.44) at TTS = 90 days, and 2.29 (95% CI: 0.63–8.31) at TTS = 120 days (Fig. 2). Stratification of HER2 + patients by HR status showed no substantial difference in mortality risk associated with TTS (*data not shown*). In contrast, estimated BCSM risk for HR−/HER2− patients showed a much smaller linear increase in sHR of approximately 0.04 weekly **(Supplemental Fig. 1)**, with sHR estimates not significantly different from 1 across the examined range of TTS, also potentially due to small event numbers (Fig. 2).

**Figure 3:**
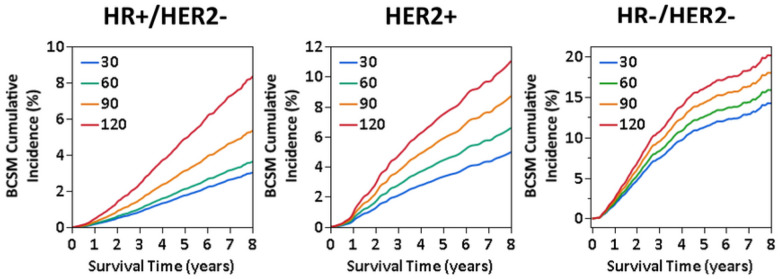
Adjusted cumulative incidence function (CIF) of breast cancer-specific mortality (BCSM) in HR+/HER2−, HER2+, and HR−/HER2− locoregional breast cancer patients at specified TTS points (30, 60, 90, and 120 days) in a SEER-Medicare cohort.

Estimates from the models were then conditioned on a set of the most common covariates within each subtype to calculate the cumulative incidence function (CIF) for TTS at 30, 60, 90, 120 days. Consistent with the observed exponential nature, adjusted 5-year BCSM cumulative incidence was 0.4% (2.1%; 95% CI: 1.8–32.5%) at TTS = 60 days, 1.4% at TTS = 90 (3.1%; 95% CI: 2.4–4.1%), and 3.1% (4.9%; 95% CI: 3.0–8.1%) higher at TTS = 120 compared to TTS = 30 days. Adjusted 8-year BCSM cumulative incidence was accordingly 0.6% (3.0%; 95% CI: 2.6–3.5%) at TTS = 60 days (3.6%; 95% CI: 3.0–4.4%), 2.4% at TTS = 90 (5.4%; 95% CI: 4.2–6.8%),5.4% (8.4%; 95% CI: 5.2–13.3%) higher at TTS = 120 compared to TTS = 30 days (Fig. 3A). In contrast, a difference of approximately 1.4% in adjusted BCSM cumulative incidence at 5-years between each 30-day point in TTS was observed, leading to a difference of 4.1% between TTS = 30 days (3.3%, 95% CI: 2.4–4.5%) and TTS = 120 days (7.4%, 95% CI: 2.0–27.8%) in HER2 + and a 1.9% difference at 8-years (TTS = 30 days: 4.8%, 95% CI: 3.4–6.7%; TTS = 120 days: 10.6%, 95% CI: 3.3–34.1%; Fig. 3B). Similarly, in HR−/HER2− patients, a difference of approximately 1.6% in 5-year adjusted BCSM cumulative incidence was observed between each 30-day point in TTS, or a 4.8% total difference between TTS = 30 days (11.3%, 95% CI: 9.1–614.0%) and TTS = 120 (16.1%, 95% CI: 6.6–39.1%), and 2.0% (TTS = 30 days: 14.2%, 95% CI: 11.6–17.5%; TTS = 120 days: 20.2%, 95% CI: 9.5–42.7%) in 8-year, resulting in a 6% difference between TTS = 30 days (14.2%, 95% CI: 11.6–17.5%) and TTS = 120 days (20.2%, 95% CI: 9.5–42.7%; Fig. 3C).

## Discussion

Our study is the first to provide dynamic insight into subtype-specific differential patterns of BCSM risk associated with TTS. Through a robust statistical approach, capturing flexible daily estimates of BCSM risk, rather than broadly grouped, discrete TTS intervals^2,6,12,13^, we found that patients with HR+/HER2− breast cancer experienced rapid exponential trajectory of TTS-associated mortality risk, as opposed to the slower linear growth seen for patients with HER2 + and HR−/HER2− breast cancer. The adjusted TTS-associated BCSM cumulative incidence in HR+/HER2− patients was reflected by increasingly larger gaps, with approximately 5% higher 8-year mortality in patients with TTS = 120 days compared to 30 days (3.0% vs. 8.4%). This is important especially since patients with TTS beyond 60 days are continually exposed to growing risk across the follow-up period. Our data showed growing BCSM risk in all subtypes with increasing TTS, albeit non-significant in HR−/HER2− patients, emphasizing the benefit of timely surgery after biopsy diagnosis, consistent with the CoC’s recent quality measure for surgery within 60 days of diagnosis for non-neoadjuvant Stage I-III patients^10^.

The observed difference by subtype was unexpected based on the prevailing view of favorable prognosis for HR+/HER2− disease and less favorable prognosis for HER2 + and HR−/HER2− breast cancer^19^. Death from breast cancer is primarily the result of metastatic outgrowth of disseminated cells in distant organs, thus provoking the question as to whether metastatic dissemination occurs differently by subtype over the duration of TTS. Breast cancer dissemination is proposed to occur by two key mechanisms: 1) linear progression in which cancer cells and the tumor microenvironment gradually acquire a phenotype conducive to metastasis or 2) parallel progression through early dissemination of inherently metastatic cancer cells in response to an angiogenic switch^20,21^. In this light, TTS may provide extra time for progressive phenotypic or microenvironment changes to occur. Mathematical simulation indicates that the natural breast tumor growth rate is relatively slow, taking approximately 1.7 years for a 1 cm tumor to double in size^22^. Thus, tumor size upstaging of T1N0M0 patients^13^ as well as exponential BCSM risk in HR+/HER2− after a brief 42-day period following diagnosis may suggest the possibility of accelerated disease progression beyond the rate of natural linear progression after diagnosis^13^. On the other hand, TTS-associated mortality could reflect an intrinsic metastatic subpopulation within the highly heterogeneous HR+/HER2− subtype, since both Oncotype recurrence risk score and percentage of estrogen receptor/progesterone receptor positivity contribute to substantial prognostic diversity even in early stage disease^23–25^. Such differences should be further investigated to determine their role in TTS-associated outcomes. Along similar lines, adjuvant chemotherapy may potentially impact TTS-associated BCSM risk by attenuating the likelihood that cancer cells disseminated during the diagnosis-to-surgery timeframe survive. Indeed, only 21% of HR+/HER2− patients in our cohort received systemic chemotherapy due to expected insufficient response^26^, as compared to 68% and 56%, in HER2 + and HR−/HER2− breast cancer, respectively (Table 1). Since subtype as well as Oncotype score are critical determinants of treatment course, we are unable to disentangle the potential effects of chemotherapy in the current study. Finally, a biologic basis for the observed increase in BCSM with longer surgical delays remains unknown and further studies are warranted, particularly to shed light on the high burden of TTS-associated mortality risk in HR+/HER2− patients.

The primary strength of this study lies in robust statistical modelling to identify the dynamic nature of mortality risk associated with TTS. Integrating the novel approach for propensity score calculation for non-parametric continuous variables developed by Imai and colleagues^16–18^ to adjust for socio-demographic and clinical characteristics, Fine-Gray competing risk survival analysis delineated non-linear TTS-associated risk of BCSM in HR+/HER2− patients, which may not be visible when fixed effect sizes across TTS monthly or bi-monthly increments are compared. Limitations of this study include the potential for confounders outside the scope of, or with incomplete reporting in, the databases (e.g. clinical staging, Ki67 status). Additionally, the cohort is composed of an elderly population with Medicare coverage and the sample size of patients with HER2 + or HR−/HER2− subtype, which typically make up a greater proportion of younger patients^27^, is limited, resulting large confidence bounds. Thus, further studies of differences in TTS-associated BCSM risk by subtype in younger women as well as across more diverse insurance types is recommended. Lastly, a plausible biologic mechanism to adequately explain such rapid mortality risk increases within a relatively short period of surgical delay following diagnosis is currently unavailable. Further study to elucidate the underlying reasons for TTS-associated mortality risk, including delay of adjuvant therapies, biologic changes^28^, or natural disease progression, is warranted.

## Conclusion

This study identified that the association between surgical delay and BCSM risk varies by tumor subtype, with a rapid exponential increase in risk in HR+/HER2− patients and lesser linear increases in patients with HER2 + or HR−/HER2− breast cancer. Prevention of surgical delays holds the potential to improve survival outcomes for patients with locoregional breast cancer across multiple tumor subtypes.

## Figures and Tables

**Table 1.** Distribution of TTS by cohort demographic and clinical characteristics within subtypes

	HR+/HER2−		HER2+		HR−/HER2−	
	n (%)	Median (Q1–Q3)	n (%)	Median (Q1–Q3)	n (%)	Median (Q1–Q3)
	28,332 (100)	29 (21–42)	3,226 (100)	29 (20–41)	2,690 (100)	29 (20–42)
**Age**						
*<70*	7,766 (27.4)	31 (21–44)	919 (28.5)	29 (20–42)	637 (23.7)	29 (20–42)
*70– 74*	8,678 (30.6)	29 (21–42)	914 (28.3)	29 (20–41)	782 (29.1)	29 (20–42)
*75– 79*	6,254 (22.1)	29 (21–42)	693 (21.5)	28 (20–40)	595 (22.1)	29 (20–43)
*80–84*	3,687 (13.0)	28 (20–41)	439 (13.6)	29 (20–41)	383 (14.2)	28 (20–41)
*85+*	1,947 (6.9)	28 (20–42)	261 (8.1)	28 (20–40)	293 (10.9)	29 (21–40)
**Race/Ethnicity**						
*White*	25,206 (89.0)	29 (21–42)	2,775 (86.0)	28 (20–41)	2,234 (83)	28 (20–41)
*Black*	1,526 (5.4)	34 (23–48)	230 (7.1)	31 (22–51)	320 (11.9)	36 (23–51)
*Other*	1,600 (5.6)	33 (22–44)	221 (6.9)	30 (21–43)	136 (5.1)	29 (20–43)
**Charlson Comorbidity Index**						
*0*	16,102 (56.8)	29 (21–42)	1,738 (53.9)	28 (19–40)	1,411 (52.5)	28 (20–41)
*1*	6,820 (24.1)	29 (21–42)	794 (24.6)	29 (20–41)	650 (24.2)	28 (20–42)
*2+*	5,410 (19.1)	31 (21–45)	694 (21.5)	32 (22–44)	629 (23.4)	31(21–45)
**Year of Diagnosis**						
*2010*	3,021 (10.7)	27 (19–38)	370 (115)	25 (17–37)	348 (12.9)	28 (19–40)
*2011*	3,173 (11.2)	28 (19–40)	367 (11.4)	27 (18–36)	338 (12.6)	27 (19–41)
*2012*	3,407 (12.0)	29 (20–41)	397 (12.3)	28 (18–38)	371 (13.8)	27 (19–37)
*2013*	3,553 (12.5)	29 (20–42)	397 (12.3)	28 (20–42)	324 (12.0)	29 (20–43)
*2014*	3,564 (12.6)	29 (21–42)	424 (13.1)	30 (21–42)	345 (12.8)	28 (21–42)
*2015*	3,780 (13.3)	31 (22–43)	463 (14.4)	29 (21–42)	311 (11.6)	32 (22–43)
*2016*	3,998 (14.1)	32 (22–44)	417 (12.9)	31 (22–43)	321 (11.9)	31 (21–45)
*2017*	3,836 (13.5)	33 (22–47)	391 (12.1)	32 (24–46)	332 (12.3)	31(22–43)
**SEER Stage**						
*Local*	22,874 (80.7)	29 (21–42)	2,342 (72.6)	28 (20–41)	2,152 (80.0)	29 (20–42)
*Regional lymph node involvement*	5,458 (19.3)	30 (21–44)	884 (27.4)	29 (20–42)	538 (20.0)	29 (20–44)
**Histology**						
*Ductal*	21,086 (74.4)	29 (21–42)	2,849 (88.3)	28 (20–41)	2,312 (85.95)	29 (20–42)
*Lobular*	5,736 (20.2)	32 (22–44)	282 (8.7)	33 (23–49)	104 (3.87)	29 (19–45)
*Other*	1,510 (5.3)	29 (21–41)	95 (2.9)	30 (18–43)	274 (10.19)	31 (21–44)
**Grade**						
*1*	9,615 (33.9)	29 (21–42)	240 (7.4)	28 (20–40.5)	86 (3.2)	28 (19–43)
*2*	1,4745 (52.1)	30 (21–43)	1,244 (38.6)	30(20–42)	661 (24.57)	31 (21–43)
*3 or 4*	3,972 (14.0)	28 (20–42)	1742 (54)	28 (20–41)	1,943 (72.2)	28 (20–41)
**Type of Surgery**						
*Breast Conserving*	19,730 (69.6)	29 (21–41)	1,775 (55)	28 (20–40)	1,602 (59.6)	28 (21–41)
*Mastectomy*	7,179 (25.4)	29 (20–43)	1,265 (39.2)	28 (20–41)	982 (36.5)	29 (19–43)
*Mastectomy w/Reconstruction*	1,423 (5.0)	45 (33–61)	186 (5.8)	44 (32–60)	106 (3.9)	41 (27–60)
**Chemo/Targeted Therapy**						
*No*	22,354 (78.9)	29 (21–42)	1,030 (31.9)	29 (21–42)	1,171 (43.5)	30 (21–43)
*Yes*	5,978 (21.1)	29 (20–42)	2,196 (68.1)	29 (20–41)	1,519 (56.5)	28 (20–41)
**Radiation**						
*No*	9,714 (34.3)	31 (21–46)	1,258 (39)	30 (20–45)	903 (33.6)	30 (20–44)
*Yes*	18,618 (65.7)	29 (21–41)	1,968 (61)	28 (20–40)	1,787 (66.4)	29 (20–41)
**HR status**						
*Negative*			1,200 (37)	29 (20–42)	2,690 (100)	
*Positive*	28,332 (100)		2,026 (63)	28 (20–41)		
**Cause of Death**						
*Breast Cancer*	900 (3.2)	28 (20–44)	210 (6.5)	29 (20–41)	342 (12.7)	29 (19–42)
*Other*	2,800 (9.9)	28 (20–42)	334 (10.4)	30 (20–42)	324 (12.0)	28 (20–43)
*Censored*	24,632 (86.9)	30 (21–42)	2,682 (83.1)	29 (20–41)	2,024 (75.3)	29 (21–42)

**Table 2:** Adjusted risk of breast cancer-specific mortality at weekly points of TTS by subtype

	HR+/HER2−		HER2+		HR−/HER2−	
	sHR	95% CI	sHR	95% CI	sHR	95% CI
**TTS (days)**						
14	1.15	(0.99–1.31)	1.06	(0.79–1.42)	1.07	(0.85–1.34)
21	1.05	(0.97–1.13)	0.97	(0.84–1.13)	1.00	(0.89–1.13)
28	1.00	(0.99–1.02)	0.99	(0.96–1.02)	1.00	(0.98–1.02)
35	1.00	(0.97–1.03)	1.04	(0.97–1.12)	1.01	(0.96–1.07)
42	1.03	(0.96–1.11)	1.11	(0.96–1.28)	1.04	(0.93–1.16)
49	1.08	(0.98–1.19)	1.2	(0.98–1.46)	1.07	(0.92–1.25)
56	**1.15**	**(1.02–1.29)**	**1.28**	**(1.02–1.64)**	1.11	(0.91–1.34)
63	**1.24**	**(1.08–1.42)**	**1.38**	**(1.02–1.87)**	1.14	(0.91–1.44)
70	**1.36**	**(1.16–1.59)**	**1.48**	**(1.02–2.13)**	1.18	(0.89–1.56)
77	**1.49**	**(1.25–1.80)**	**1.59**	**(1.00–2.51)**	1.22	(0.86–1.72)
84	**1.64**	**(1.32–2.02)**	1.69	(0.97–2.94)	1.26	(0.83–1.90)
91	**1.83**	**(1.42–2.37)**	1.8	(0.92–3.53)	1.30	(0.79–2.14)
98	**2.03**	**(1.50–2.74)**	1.92	(0.85–4.35)	1.34	(0.73–2.45)
105	**2.26**	**(1.59–3.22)**	2.04	(0.78–5.35)	1.38	(0.68–2.82)
112	**2.50**	**(1.67–3.78)**	2.15	(0.71–6.51)	1.42	(0.63–3.21)
119	**2.79**	**(1.75–4.45)**	2.71	(0.64–8.06)	1.46	(0.58–3.71)

## Abbreviations

HR+: Hormone receptor-positive
HR−: Hormone receptor-negative
HER2: Human epidermal growth factor receptor 2
TTS: Time from biopsy to surgery
BCSM: Breast cancer specific mortality
HR: Hazard ratio
sHR: Subdistribuition hazard ratio
IPW: Inverse propensity score weights
SEER: Surveillance, Epidemiology, and End Results
